# Supplementary material for: Phagocyte Chemoattraction Is Induced through the Mcp-1–Ccr2 Axis during Efferocytosis
Source: Cells. 2021 Nov 10;10(11):3115. doi: 10.3390/cells10113115 (PMC8620886; doi:10.3390/cells10113115)
Supplement: Supplementary file 1 [file cells-10-03115-s001.zip › cells-1434864-SM-figures-revised.pdf]

Supplementary Figures

Phagocyte chemoattraction is induced through the Mcp-1-Ccr2 axis during efferocytosis

Sang-Ah Lee [1,2], Deokhwan Kim [1,2], Chanhyuk Min [1,2], Byeongjin Moon [1,2], Juyeon Lee [1,2], Hyunji Moon [1,2], Susumin Yang [1,2], Chang Sup Lee[3], Gwangrog Lee[1,2], and Daeho Park [1,2,*]

[1] *School of Life Sciences, Gwangju Institute of Science and Technology, Gwangju 61005, Republic of Korea; sanga03@gist.ac.kr (S.-A.L.); po7322@gist.ac.kr (D.K.); alscksgur@gist.ac.kr (C.M.); byeongjinmoon@gist.ac.kr (B.M.); iris260@gist.ac.kr (J.L.); hjmoon311@gist.ac.kr (H.M.); susuminy@gist.ac.kr (S.Y.); gwangroglee@gist.ac.kr (G.L.); daehopark@gist.ac.kr (D.P.)*

[2] *Laboratory of Cell Mechanobiology, Gwangju Institute of Science and Technology, Gwangju 61005, Republic of Korea*

[3] *College of Pharmacy and Research Institute of Pharmaceutical Sciences, Gyeongsang National University, Jinju, 52828, Republic of Korea; changsup@gnu.ac.kr (C.L.)*

***Correspondence to**

Daeho Park

School of Life Sciences

Gwangju Institute of Science and Technology

123 Cheomdangwagi-ro, Buk-gu

Gwangju 61005, Korea

Tel.: 82-62-715-2890

Fax: 82-62-715-2484

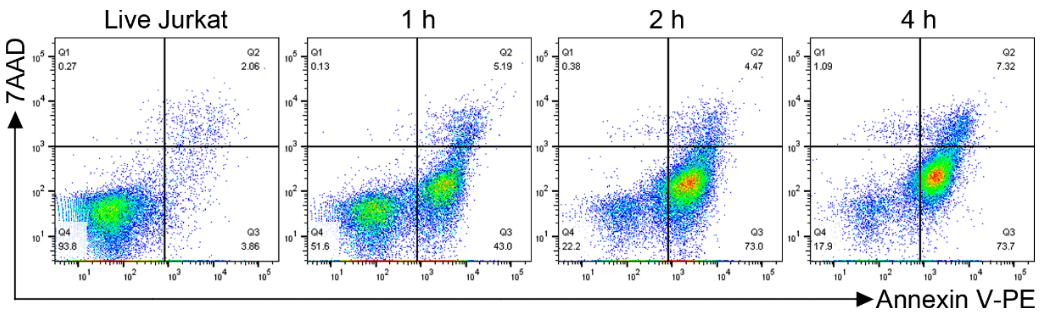

Figure S1. The percentages of apoptotic Jurkat cells after UV irradiation

Jurkat cells in DPBS were irradiated with 100 mJ/cm^2 ultraviolet-C (UVC) and then incubated in complete RPMI medium at 37°C for the indicated times. After that, the cells were stained with 7AAD and Annexin V and analyzed by flow cytometry.

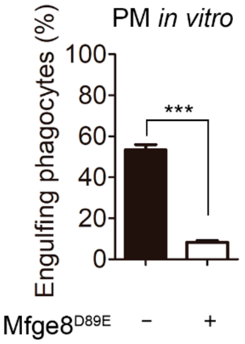

Figure S2. Mfge8^{D89E} abrogates efferocytosis by peritoneal macrophages

Peritoneal macrophages derived from wild-type mice were incubated with TAMRA-labeled apoptotic thymocytes in the presence and absence of Mfge8^{D89E} for 15 min. Then, unbound apoptotic cells were washed with ice-cold DPBS and peritoneal macrophages engulfing apoptotic cells were analyzed by flow cytometry. Data are shown as the mean ± SEM. ***P<0.001. PM, peritoneal macrophages.

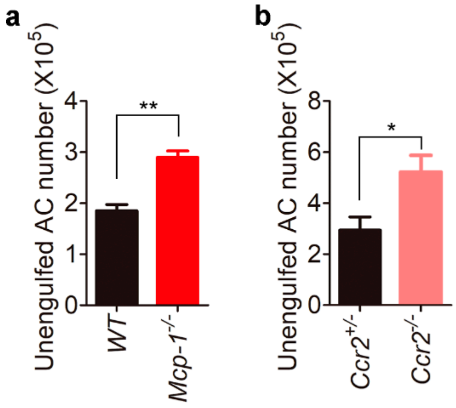

Figure S3. Clearance of apoptotic cells is impaired in *Mcp-1*$^{-/-}$ and *Ccr2*$^{-/-}$ mice

(**a**) TAMRA-labeled apoptotic thymocytes were intraperitoneally injected into *WT* or *Mcp-1*$^{-/-}$ mice. Two hours after injection, peritoneal exudates were collected, stained with an anti-F4/80 antibody, and analyzed by flow cytometry. The number of TAMRA-positive and F4/80-negative cells per counting bead was determined. (**b**) CellTracker-labeled apoptotic thymocytes were intraperitoneally injected into *Ccr2*$^{+/-}$ or *Ccr2*$^{-/-}$ mice. Two hours after injection, peritoneal exudates were stained with an anti-F4/80 antibody and analyzed by flow cytometry. The number of CellTracker-positive and F4/80-negative cells per counting bead was determined. Data are shown as the mean ± SEM. *P<0.05, **P<0.01.
